# When the microbiome shapes the host: immune evolution implications for infectious disease

**DOI:** 10.1098/rstb.2023.0061

**Published:** 2024-05-06

**Authors:** Mark A. Hanson

**Affiliations:** Centre for Ecology and Conservation, University of Exeter, Cornwall, TR10 9FE, UK

**Keywords:** host–microbe interactions, host–pathogen, reactive oxygen species, antimicrobial peptide, host defence peptide, microbiota

## Abstract

The microbiome includes both ‘mutualist’ and ‘pathogen’ microbes, regulated by the same innate immune architecture. A major question has therefore been: how do hosts prevent pathogenic infections while maintaining beneficial microbes? One idea suggests hosts can selectively activate innate immunity upon pathogenic infection, but not mutualist colonization. Another idea posits that hosts can selectively attack pathogens, but not mutualists. Here I review evolutionary principles of microbe recognition and immune activation, and reflect on newly observed immune effector–microbe specificity perhaps supporting the latter idea. Recent work in *Drosophila* has found a surprising importance for single antimicrobial peptides in combatting specific ecologically relevant microbes. The developing picture suggests these effectors have evolved for this purpose. Other defence responses like reactive oxygen species bursts can also be uniquely effective against specific microbes. Signals in other model systems including nematodes, *Hydra*, oysters, and mammals, suggest that effector–microbe specificity may be a fundamental principle of host–pathogen interactions. I propose this effector–microbe specificity stems from weaknesses of the microbes themselves: if microbes have intrinsic weaknesses, hosts can evolve effectors that exploit those weaknesses. I define this host–microbe relationship as ‘the Achilles principle of immune evolution’. Incorporating this view helps interpret why some host–microbe interactions develop in a coevolutionary framework (e.g. Red Queen dynamics), or as a one-sided evolutionary response. This clarification should be valuable to better understand the principles behind host susceptibilities to infectious diseases.

This article is part of the theme issue ‘Sculpting the microbiome: how host factors determine and respond to microbial colonization’.

## 1. Introduction

Animals live in a microbial world. Incredibly complex networks of microbe communities interact with each other both within and outside animal hosts. These relationships span a spectrum from mutualistic symbiosis, to commensalism, to pathogenic growth. This places a pressure on animals to control a vast array of potential microbial colonizers, ensuring beneficial ones are encouraged while pathogenic ones are suppressed. The innate immune system is key in this arena, as innate immune responses are the first line of defence against infection. This creates an interesting set of evolutionary optima for both microbes and their hosts: those microbes that have intimate relationships with their hosts should coevolve to avoid activating or being damaged by host immune responses and may even protect hosts themselves. Meanwhile, hosts should ideally evolve immune responses that remain inactive in the presence of mutualist microbes, but are selectively active against pathogens. Hosts should also have immune response repertoires that are both induced rapidly enough to be effective in controlling pathogens, but will largely avoid harming mutualist microbes. The mechanisms of the host immune response are multi-faceted, providing an arena for evolution that can be engaged at all levels of immune activation and signal communication.

In this review, I highlight the ways that microbes activate host immune responses and present a logic for how the activation of animal innate immunity has evolved. I also discuss recent findings of innate immune effector specificity and evolution that have changed the way we view terminal products of immune signalling. Recent work in invertebrates, particularly *Drosophila* fruit flies, has allowed a dissection of innate immune principles that are difficult to study in mammals owing to the co-activation of adaptive immune responses upon infection. The question has now become whether vertebrate effectors are similarly precise, with some evidence already pointing in this direction. This revised view of the logic guiding immune evolution helps understand the selective pressures that drive host–microbe interactions, and should inform on principles behind infectious disease issues of global importance.

## 2. Information bottlenecks lead to generalist immune activation

**Figure 1.**
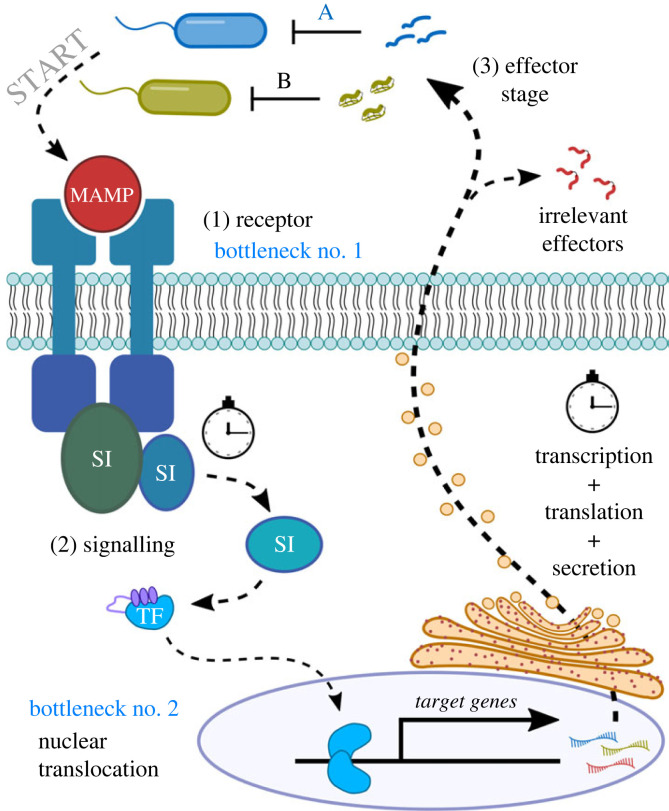
The innate immune response can be divided into three stages: (1) at the receptor stage, a microbe-associated molecular pattern (MAMP) is sensed by receptors, which transmit that information through conformation changes that recruit signalling intermediates (SIs). Pathways can also be activated by damage signals; (2) signalling is then accomplished by proteolytic cascades that ultimately activate and translocate immune transcription factors (TFs) to the nucleus, where they enhance the expression of target genes. At this stage, hundreds of target genes are induced. The defence response must then transcribe target genes, translate their messenger RNAs, and secrete the encoded immune effectors rapidly enough to quell a potential infection; and (3) the effector stage reflects the realized immune response: immune signalling culminates in the production of molecules that can actually change the course of a potential infection (e.g. antimicrobial peptides). Importantly, there are major information bottlenecks in this process. For instance, information (MAMPs) sensed by receptors is not very precise, and so many pathogens with minimal relatedness all trigger the same signalling cascades. Moreover, information transmitted through signalling intermediates is again simplified through just one or a few cascades or transcription factors. As a result, the receptor and signalling stages lack the specificity needed to mount gene induction precise enough to be useful only against the pathogen that initiated the response—coinfections are likely in natural settings anyways (see discussion by Franz *et al*. [1]). Consequently, there is little benefit to precision at the recognition and signalling stage. Instead, trade-offs between precision and having an effective response time promotes a rapid ‘all-hands-on-deck’ principle to immune activation. However, recent studies suggest the effector stage is not similarly generalist. Instead, specific effectors are uniquely important for combatting specific pathogens (e.g. effector A versus pathogen A). As such, many of the effectors produced by generalist immune activation are largely irrelevant to the initiating pathogen(s).

Innate immune signalling pathways can be divided into three stages: receptor, signalling intermediate and effector (figure 1). I will focus first on the receptor and intermediate stages.

At the receptor stage, microbe-associated molecular patterns (MAMPs) are sensed by host molecules. Alternately, the host can sense damage signals independent of a specific infection, and trigger immune responses in a microbe-agnostic way. MAMPs are typically broadly conserved molecules common to many microbes or infectious processes: for instance, bacterial peptidoglycan or lipopolysaccharide, fungal glucans or even the presence of intracellular nucleic acids indicating damage or infection [2–4]. Depending on the tissue where the receptor is activated, MAMP-binding sends a message: ‘microbes are present in places they shouldn't be’. Upon MAMP-binding, receptors undergo a conformational change, recruiting and activating signalling intermediates, which communicate and amplify information to downstream targets. These cascades can be regulated at multiple levels by processes such as receptor localization, ubiquitination or phosphorylation, all ways that cells can inhibit or enhance the activity of immune signalling [5–7]. Ultimately, the cascade converges to activate transcription factors (TFs) [8,9], master regulators of gene expression. Most immune pathways result in the activation of just one or a select few TFs. This bottleneck in information flow ensures that a relevant subset of the host immune repertoire is induced upon infection. However, this process is fairly generalist, as entire classes of microbes can share membrane components or other MAMPs, which activate the same cascade. For instance, diaminopimelic acid (DAP)-type peptidoglycan is common to all Gram-negative bacteria and also some Gram-positive bacteria, and triggers the expression of hundreds of genes regulated by the insect Imd pathway, a cascade with homology to vertebrate Toll-like receptor (TLR), tumour necrosis factor receptor-alpha (TNFR-α) and nucleotide-binding oligomerization domain-like (NOD)-like receptor pathways [10].

Innate immune signalling therefore passes information through bottlenecks: a few broadly conserved MAMPs activate the cascades that are present, and these cascades converge on just one or a few TFs. This bottleneck regulatory approach is also part of the hourglass model of ‘evo-devo’ and ‘evo-immuno’ dynamics, discussed in [11]—in immunology, they may reflect a trade-off between producing a rapid defence or a specific one (box 1).

Box 1. **Evolutionarily, speed is preferred over precision. This probably reflects the evolutionary limits of selection to maintain diverse genes or isoforms, and/or optimization of metabolic efficiency. In *Drosophila*, females already suffer from trade-offs between reproductive success and inducing a rapid defence response [12]. To communicate pathogen-specific information even for just 100 bacteria types, hosts would need to increase investment in immune surveillance to regulate 100 unique receptor isoforms—not to mention hundreds of corresponding signalling intermediates and transcription factors—to enable pathogen-specific immune response programmes. This would probably be inordinately costly, and pathogen evolution or genetic drift could readily undermine such specific cascades. The use of generally relevant receptors and relatively few pathways may thus optimize trade-offs between immunity and other physiological needs. This is enabled by focusing on broadly conserved MAMPs that are essential for microbes to function. The consequence, however, is that whole suites of genes are co-expressed under the regulation of cascades with few receptors and transcription factors, removing the potential for precise transmission of information.**

### (a) Can hosts selectively activate immune responses only in the presence of pathogens?

Given these bottlenecks in information presented from receptors through to transcription factors, there is little evidence or apparent mechanism, to suggest that hosts can avoid immune activation by mutualist microbes: the MAMPs of mutualists are the same as those of pathogens.

As such, perhaps the key difference between mutualists and pathogens in terms of immune activation is essentially the microbe's predisposition to ‘do as it's told’. For instance, *Pectobacterium Ecc15* bacteria encode virulence factors that perforate insect guts, which enables their escape into the haemolymph after intestinal infection [13]. Meanwhile, *Acetobacter* bacteria are common mutualist microbes of the *Drosophila* gut, and typically remain in the gut compartments [14]. The reason *Pectobacterium* can cause a systemic immune response upon oral infection is partly the damage it does to the host gut, but more importantly, it invades compartments where immune surveillance and potential for systemic response is high, such as the body cavity [8]. Mutualists like *Acetobacter* do not puncture the gut epithelium and enter the body cavity in the same way, preventing local or systemic immune hyperactivation. In this way, host immune signalling is uniquely activated by the presence of pathogens, but less so by mutualists, despite the same immune architecture sensing MAMPs common to both bacteria [5]. This physical separation of mutualist microbes from the surveilling elements of the systemic immune response is a common strategy of insects with obligate symbionts, which have evolved specialized immune-competent tissues in which they house their beneficial microbes [14–16].

Taken together, intrinsic microbe properties do not readily delineate mutualist from pathogen at their surface. Instead, discrimination relies on host–microbe interactions themselves: hosts surveil for microbes in compartments where microbes should not be, and pathogens are the ones that find ways to enter those compartments—with some exceptions (e.g. [17]). Serious inflammatory responses are activated only when microbes are out of bounds, in places such as the basal lamina under epithelia, or in more serious infections, the host circulatory system (systemic infection).

## 3. Specificity of the immune response is accomplished by effectors

The fact that immune pathway recognition and activation is so generalist led to the assumption that the suite of co-expressed immune effectors should be similarly generalist [18,19]: here, I will call this the ‘additive model of immune defence’. In the additive model, all effectors contribute to defence against all pathogens, and a successful immune response relies on the collective action of all effectors. Implicit in this assumption is that most effectors contribute meaningfully to any given infection. Importantly, this model of immune defence is supported by *in vitro* experiments: for instance, host antimicrobial peptides (AMPs) are immune effectors that are induced hundreds of fold upon infection and kill a broad range of microbes *in vitro* [18,19]. Bursts of reactive oxygen species (ROS) are also common across animals, and have broad microbicidal activity *in vitro* [20,21]. In the additive model, all AMPs or immune ROS are expected to contribute to microbe-killing, and if the production of any one of these effectors is reduced, there should be sufficient redundancy among effector mechanisms that the consequence of losing just one effector should be minimal. However, there are also cases where host resistance may be at a fine balance, where loss of even a single effector in the additive model could leave the host highly susceptible to infection as its defence capacity drops below a theoretical minimum microbicidal activity needed for pathogen suppression.

This additive model of defence was implicit in next-generation sequencing studies of the immune response. Observations that tens to hundreds of genes are differentially expressed upon a given infection could imply that every differentially expressed gene candidate has a partial role in the defence response to that infection, which could be revealed if one bothered to look. Many lines of evidence led to this interpretation [18], however, these assumptions of the past are now being overturned. Here I will argue that this past interpretation is owing to misdirection by evolutionary spandrels [22]: byproducts of natural selection that are not related to the exact question at hand. As outlined above, the evolutionary reason that so many genes are co-expressed is not necessarily because each has an important role to play for every infection, but because information is transmitted through bottlenecks, leaving little room for precision in the inducible immune response. As such, a suite of genes is induced upon certain infections that may not confer any selective advantage at all, and instead represents an incredible metabolic waste. This idea, while seemingly unintuitive, has now been demonstrated robustly in *Drosophila* fruit flies, and seems likely to be true of animals more generally.

### (a) Immune effector specificity is key in host–pathogen interactions

While many processes are differentially regulated upon infection, immune defence itself is accomplished by the production of molecules that directly damage pathogens: the effector stage. Recent loss-of-function genetic studies in *Drosophila* have repeatedly found that loss of single effector genes or mechanisms creates striking susceptibilities to specific microbes. For instance, the insect melanization reaction produces an ROS burst similar to ROS responses in vertebrate immune cells downstream of integrins, TLRs or TNFR signalling [20,21]. It is therefore intriguing that the primary fly defence against *Staphylococcus aureus* relies on the ROS burst from the *Drosophila* melanization reaction, while other immune mechanisms such as AMPs or phagocytosis by macrophage-like cells are less essential [21,23]. This parallels findings from experimental evolution studies in *Caenorhabditis elegans* nematodes, where defence against *S. aureus* is evolved through upregulation of ROS responses, both by the nematodes themselves [24], and by coevolving mutualist microbes [25]. Recent work in human macrophages has shown that phagocytosis is ineffective in suppressing *S. aureus*. Indeed, *Staphylococcus aureus* is greatly affected by the ROS burst accompanying phagocytosis, with the unintended consequence that the few surviving bacteria can display improved ability to resist antibiotic treatment [26]. These findings collectively suggest that, out of many potential immune mechanisms, *S. aureus* is most significantly impacted by ROS bursts, and ROS is among the most effective *S. aureus* killing tools in animals. Therapeutics that bolster existing macrophage ROS responses could therefore be a highly effective strategy to kill *S. aureus*, rather than simply arrest its cellular respiration.

Another fundamental defence mechanism is the phagocytosis of parasites and pathogens. For instance, *Mycobacterium abscessus* is combatted by certain types of phagocytes, with the caveat that phagocytosis of *M. abscessus* may even protect the bacteria from the host AMP response [27,28]. Indeed, many Gram-positive bacteria seemingly ignore host AMPs [23,29], which could also depend on their thick cell wall made of peptidoglycan and techoic acids [30]. As such, mechanisms of defence like ROS bursts or phagocytosis may be specifically useful against Gram-positive bacteria because alternatives like AMPs are ineffective for one reason or another. While the picture is less clear for what defence(s) are effective against the microsporidian parasite *Tubulinosema ratisbonensis*, phagocytosis is also uniquely important for host survival upon systemic infection [31]. More generally, eukaryotic parasites tend to be combatted by cellular responses, most likely because any molecular responses that immune cells use are made more effective by concentrating their effects through trapping parasites in networks of immune cells [32,33]. Exceptions to this model of anti-parasite defence exist, including the use of toxins that can specifically target the parasite but spare the host. Such parasite-specific toxins can be encoded by defensive symbionts [34,35], giving the host an accessory genome with defence molecules that confer an advantage upon exposure to parasites [36].

### (b) Antimicrobial peptides specificity against Gram-negative bacteria and fungi

The most striking results of effector–microbe specificity have perhaps come from recent work in *Drosophila* dissecting the individual and combinatory contributions of effector peptides to defence. This work has been particularly surprising for a number of reasons. First, when the seven classic fly AMP gene families are deleted, there is almost no effect seen upon infections by many Gram-positive bacteria (including *S. aureus*, *Staphylococcus pneumoniae*, *Enterococcus faecalis, Listeria monocytogenes, Bacillus subtilis, M. abscessus* and *Lactiplantibacillus plantarum*) [23,27,29,30].

Another surprising finding has been the incredible importance of single AMPs in defence against specific Gram-negative bacteria. Two groups collectively demonstrated that the fly AMP Diptericin specifically defends against the opportunistic pathogen *Providencia rettgeri*. First, Unckless *et al*. [37,38] detected a single-nucleotide polymorphism in the fly *Diptericin A* gene (*DptA*) through genome-wide association study (GWAS). Flies encoding an arginine variant at DptA residue 69 were far more susceptible to infection than flies encoding serine (DptA polymorphism S69R). Later, Hanson *et al*. [23] systematically deleted genes of AMP families in *D. melanogaster*, finding that loss of *Diptericins* alone was sufficient to explain the susceptibility of immune deficient animals lacking the Imd pathway. Incredibly, deletion of eight other AMP genes from five gene families had no impact on fly survival against *P. rettgeri*. Such specificity of AMPs for single microbes is not unique in the *Drosophila* immune response: a variety of specific AMP-bacteria associations have been demonstrated including Drosocin*-Enterobacter cloacae* [23], and Diptericin B-*Acetobacter* sp*.* ([39], discussed further below). Two studies also find similar specificity of antifungal peptides: loss of the fly AMP polypeptide gene *Baramicin A* causes host susceptibility and promotes fungal growth upon infection by *Beauveria bassiana*. Meanwhile co-deletion of the two classic fly antifungals *Drosomycin* and *Metchnikowin* had no effect alone or in combination with *Baramicin A* deletion [40]. Loss of the two *Daisho* genes also leads to a striking and specific susceptibility to *Fusarium* sp. [41]. This AMP-microbe specificity may also be found in other systems: in *Hydra* jellyfish, the AMP NDA-1 is uniquely effective against its microbiome associate *Curvibacter in vitro*, and *Curvibacter* specifically fails to colonize NDA-1-expressing tissues [42]. Oyster big Defensins similarly show drastically different inhibitory concentrations against specific microbiome-relevant bacteria [43]. Finally, a recent GWAS of Persian domestic cats found that a G49E polymorphism in the AMP Calprotectin was a major risk factor for development of ringworm fungal skin disease [44], suggesting that these highly specific and important peptide–microbe associations are not restricted to invertebrates.

Immune effectors may also promote specific defences independent of direct pathogen killing. This has been suggested for the *Drosocin* gene which encodes both the Drosocin AMP and the Buletin peptide, the latter having no known antimicrobial activity. Three studies have now identified the *Drosocin* gene as uniquely important for fly defence against *Providencia* bacteria, with one specifically identifying Buletin as the source of the effect [45–47]. A recent study of the *Baramicin A* gene also suggested a tolerance role in defence against infection by *Enterococcus faecalis* or *Metarhizium robertsii* [48]. In these cases, an intriguing hypothesis for the role of these non-killing peptides is protection against damage caused by pathogen virulence factors such as neurotoxins. Xu *et al*. [49] found this to be particularly striking for the contribution of Bomanin peptides in defence against *Aspergillus fumigatus*, while Huang *et al*. [48] found that *Baramicin A* mutants have a defective recovery rate after toxin injection, alongside evolutionary study suggesting a specific *Baramicin* peptide product can play roles in host neurology and development [50]. Such complexities are a reminder that *in vivo* roles could differ markedly from the conditions tested *in vitro*, or from assumptions based on expression profile. It should be said that, even with the advent of modern gene editing techniques, *in vitro* w­ork continues to lay the foundation for understanding the mechanisms of action of these antimicrobials. However, *in vivo* approaches appear necessary if we wish to understand the relevance of those peptides to infection in physiological conditions.

## 4. Specificity of antimicrobial peptides is a derived feature of immune evolution

These genetic studies in flies have repeatedly defied the expectations of the additive model of immune defence. Rather than many genes each providing a meaningful contribution to defence, host–pathogen interactions are regularly defined by highly important effector–microbe specificities. An outstanding question is therefore: why should specificity be so prominent? If the host evolves to have many genes induced by common regulatory networks, why should selection not favour the evolution of broadly relevant genes?

We recently provided a first experimental test to understand the evolutionary reasons behind effector–microbe specificity (Hanson *et al*. [39]). In that study, a new effector–microbe specificity was found: Diptericin B (DptB)-*Acetobacter*. There are two striking aspects to this result: (i) *DptB* is a sister gene to *DptA*, which was previously shown to have a highly specific importance against *P. rettgeri*. By infecting many species and tracking presence/absence of *Diptericins* in their genomes, we showed that genetic variation in *DptA* determines defence against *P. rettgeri*, and the same is true of *DptB* in defence against *Acetobacter*, including across species separated by approximately 50 Myr; and (ii) *Acetobacter* bacteria are typically thought of as gut mutualists, yet flies lacking *DptB* succumb to systemic infection by *Acetobacter* to the same extent as flies lacking the entire Imd NF-κB pathway. In other words: in the absence of *DptB*, *Acetobacter* is not strictly a mutualist, but rather an opportunistic pathogen. In natural settings, opportunistic systemic infections probably occur alongside parasite attack, as many *Drosophila* parasites pierce the cuticle of fly larvae that are, quite literally, swimming in *Acetobacter*. The context deciding whether a microbe is a mutualist or pathogen can therefore be defined by the presence of just a single AMP–indeed, the use of such labels is discussed in [51]. A similarly specific interaction was previously shown for a beetle peptide in control of its coevolving symbiont housed in specialized host tissue [52].

**Figure 2.**
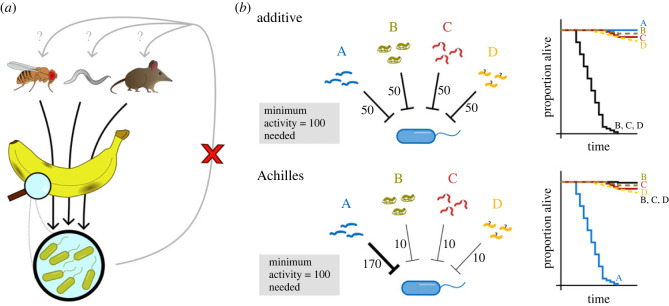
Host–pathogen dynamics explained by either the additive model or the Achilles principle. (*a*) Ecological niche determines the microbes that hosts are most likely to be exposed to over evolutionary timescales. However, from the alternate perspective, microbes will find themselves in the environment or one of many potential hosts, leading to inconsistent selective pressure from one generation to the next. As a result, a one-sided evolutionary dynamic forms, where hosts evolve immune effectors to control important and potentially infectious common microbes, but selection in the *vice versa* direction is weaker. (*b*) The host mounts multiple effector mechanisms simultaneously (A, B, C and D), and each contribute somewhat to defence. Here, those contributions are given as numbers reflecting each effector's importance to total defence activity. Importantly, the sum of all effectors must meet or exceed the minimum defence activity needed for the host to survive. Deletion of single effectors (e.g. A) or multiple effectors (B, C and D) results in different susceptibility patterns for infections governed by additive or Achilles dynamics. A continuum probably exists between additive and Achilles dynamics for most infections.

If single AMPs are key to combatting specific pathogens, this raises the question: why do those pathogens not evolve defences against these targeted attacks? Unlike the intimate relationship of host-symbiont coevolution, *Acetobacter* bacteria are common in fermenting fruits where many animals are expected to ingest them. This places a very broad evolutionary pressure on *Acetobacter*, which has to contend with the immune systems and gut defences of many animals. While fruit flies are one relevant visitor to fermenting fruits, they are unlikely to be of special importance from the *Acetobacter* perspective (figure 2*a*). Thus, flies selectively feeding on fermenting fruit have a significant selective advantage to gain by evolving an *Acetobacter*-specific defence mechanism. However, *Acetobacter* are faced with a far broader selective landscape, making it less helpful to evolve resistance to a single peptide present in only one of many potential host species. This line of reasoning has considerable implications for the development of AMPs as therapeutics [19,53]. *In vitro*, AMPs are harder to evolve resistance against than conventional antibiotics. In those experiments, peptide synergy was key [54]. However, justifying AMP therapies based on the synergy of broadly-acting AMPs *in vitro* is difficult to reconcile with recently discovered and highly specific peptide–microbe interactions *in vivo*. If a lack of important effect *in vivo* of most AMPs is true of microbial infections more broadly, this would have significant ramifications on how to develop AMPs as therapeutics. This does not somehow invalidate the potential of AMPs as inspiration for novel antibiotics. If anything, it makes their study even more important to combat antibiotic resistance: *it suggests many microbes have unique weaknesses to be exploited*.

## 5. The ‘Achilles principle’ of immune evolution

As discussed previously, the key to controlling *S. aureus* infection derived by independent model systems has been the promotion of ROS responses. Meanwhile fruit fly defences against Gram-negative bacteria frequently rely on the effects of single peptides. In both fruit flies and cats, single effector genes strongly determine susceptibility to fungal infections. Why is that?

Central to these questions is the microbe perspective. Rather than asking why the host should have microbe-specific effectors, an alternate view might be: *why do microbes have such effector-specific weaknesses?* This question instead asks which microbe traits generate specific susceptibilities. In the case of *S. aureus*, ROS responses attack iron–sulfur cluster containing proteins, including enzymes of the Krebs cycle [26]. It may be that *S. aureus* is specifically sensitive to ROS because *S. aureus* has unique protein isoforms involved in cellular respiration that are more easily disrupted by ROS. For Gram-negative bacteria like *P. rettgeri* and *Acetobacter*, different Diptericins are uniquely active against each bacterial species, yet they are expected to have the same basic mechanism of action. This suggests their specificity is instead owing to differences in their binding affinity for the microbes they target. Drosocin has many functional analogues across insect immune systems [18,55,56]. The specific importance of Drosocin against *Enterobacter cloacae* [23,47], coupled with recent data showing that Drosocin binds to bacterial ribosomes to inhibit translation [55,57], suggests a characteristic of the *Enterobacter cloacae* ribosome could make it specifically susceptible to Drosocin attack. Other mediators of Drosocin-bacteria specificity could include cell membrane components such as uptake permeases, which import Drosocin-like peptides into the cell [56,58], a prerequisite for ensuing ribosome attack.

Importantly, it is possible to hypothesize reasonable mechanisms that can explain effector–microbe specificity. This lays the groundwork for future research to demonstrate, or rule out, some of these ideas. The mere fact that effector–microbe specificity exists is key, as it suggests that host immune evolution selectively and repeatedly finds specific microbe weaknesses and exploits them. Microbes having specific weaknesses to exploit contrasts with the additive model of immune defence, which assumes that broad-acting generalist effectors are useful in defence against many microbes if there are enough of them. However, if relevant microbes have specific weaknesses that can be exploited, it instead makes sense to evolve ways to attack those precise weaknesses. In this sense, host effector evolution may sometimes favour quality over quantity.

Here I unify these observations under a new framework of immune logic: *the Achilles principle of immune evolution* (see box 2). The idea being that microbes have specific weaknesses, and hosts will evolve tailored defence strategies to target those weaknesses. This can take the form of effector optimization (as seen for fruit fly Diptericins), or changes in the speed or magnitude of a highly effective response (as seen for ROS in combatting *S. aureus* in nematodes). Importantly, the Achilles principle does not replace the additive model, but rather helps to explain host–pathogen interactions that the additive model cannot. Indeed, any given host–pathogen interaction probably exists on a continuum between purely additive or purely Achilles dynamics (figure 2*b*). The differences in selection faced by microbes or their hosts explains why hosts can evolve microbe-specific defences without invoking Red Queen host–pathogen arms races: hosts evolve Achilles principle dynamics when they are stably associated with certain microbes that have exploitable weaknesses. However, those microbes face varying hosts and so varying selective regimes, preventing coevolution from the microbe side (figure 2*a*).

Box 2. **The ‘Achilles heel’ as a narrative plot device. In storytelling, an ‘Achilles heel’ is a highly specific weakness that can be exploited to combat a powerful enemy. Examples include using silver bullets to kill werewolves, wooden stakes for vampires, or iron to repel faeries. The Achilles principle of immune evolution proposes hosts can evolve strategies to exploit microbe-specific weaknesses, akin to targeting Achilles' heel, his lone vulnerability in Greek mythology.**

## 6. Tackling resistant microbes through the lens of effector specificity

Multiple effectors can contribute additively to host defence against infection: for instance, proline-rich peptides are potentiated by pore-forming peptides [29,59], and human AMPs may synergize both to kill microbes and reduce damage to host membranes [60]. However, it is increasingly clear that single immune effectors can contribute in a profound way to defence against many infections, while others are inconsequential by comparison (table 1). These findings provide exciting new avenues to explore for therapeutics development. Indeed, use of invertebrate models has already highlighted host immune strategies uniquely effective against two ESKAPE pathogens of significant antimicrobial resistance interest (Drosocin-*Enterobacter cloacae*, ROS-*S. aureus*) [63]. The finding that single AMPs can be determinants for chronic infectious diseases like ringworm fungus in cats is also striking, as it highlights a testable mechanism of resilience against, or susceptibility to, common infectious syndromes. The recently published World Health Organization Fungal Priority Pathogens List highlighted 19 fungal pathogens of significant human importance, including *Fusarium* sp. (combatted uniquely in *Drosophila* by Daisho peptides [41]), *Aspergillus fumigatus* (suppressed by ROS, and tolerated by the action of *Drosophila* Bomanins [49]), *Candida* sp. (combatted by *Drosophila* antifungals [23,40]), and other fungi of significant concern [64]. Certain individuals are also predisposed to more subtle fungal infections including human ringworm, athlete's foot and more [64]. Fungus-driven pandemics further threaten the extinction of both amphibian and bat species [65,66]. A focus on immune effectors in resistant versus susceptible populations or species could explain why these fungal infections impact some individuals so strongly (similar to AMP-bacteria specificities affected by genetic variation [38,39]), and perhaps identify exploitable pathogen weaknesses to target with interventions and therapeutics.

**Table 1.** Key effector–microbe specificities demonstrated in the literature (There has been a major focus on effector–microbe specificity between Gram-negative bacteria or fungi and host AMPs. However, studies have identified ROS responses in flies, nematodes, and coevolved bacteria, as uniquely effective against *S. aureus*. At the same time, AMPs of fruit flies appear largely irrelevant to combatting infection by Gram-positive bacteria. As peptide activity is regularly found against Gram-positive bacteria using *in vitro* conditions, it is unclear if AMP-Gram-positive bacteria trends from *Drosophila* can be extrapolated to other organisms. However, *in vitro* activity of *Drosophila* AMPs against Gram-positive bacteria has been shown before, despite a lack of *in vivo* relevance [18].)

host	pathogen	type	effector specificity	nature of evidence	reference 1		reference 2	
fruit fly	*Staphylococcus aureus*	Gram-positive bacterium	ROS	genetic deletion, *ex vivo* correlation	Dudzic *et al*. [21]	their electronic supplementary material, figure S1	Hanson *et al*. [23]	fig. 2
	*Providencia rettgeri*	Gram-negative bacterium	DptA	GWAS, population genetics, genetic deletion, polymorphism	Unckless *et al*. [38]	paper topic	Hanson *et al*. [39]	paper topic
	*Acetobacter* sp.	Gram-negative bacterium	DptB	evolutionary genetics, genetic deletion	Hanson *et al*. [39]	paper topic		
	*Enterobacter cloacae*	Gram-negative bacterium	Drc	genetic deletion	Hanson *et al*. [23]	fig. 6	Hanson *et al*. [47]	fig. 2
	*Providencia burhodogranariea*	Gram-negative bacterium	Btn	genetic deletion, polymorphism	Hanson *et al*. [47]	fig. 2		
	*Providencia alcalifaciens*	Gram-negative bacterium	Drc or Btn	genetic deletion	Shaka *et al*. [45]	fig. 6		
	*Providencia rettgeri*	Gram-negative bacterium	Drc or Btn	genetic deletion	Shit *et al*. [45]	fig. 2		
	*Beauveria bassiana*	fungus	BaraA IM10-like	genetic deletion, *in vitro* inhibition, *in vivo* rescue	Hanson *et al*. [40]	their electronic supplementary material, figure S8		
	*Fusarium* sp.	fungus	Dso	genetic deletion, ex vivo correlation	Cohen *et al*. [41]	paper topic		
nematode + bacteria	*Staphylococcus aureus*	Gram-positive bacterium	ROS	experimental evolution, drug-based manipulation, microbe and host	King *et al*. [25]	fig. 5	Ford & King [24]	fig. 3
hydra	*Curvibacter* sp.	Gram-negative bacterium	NDA-1	*in vitro* activity specificity, *in vivo* correlational data	Augustin *et al*. [42]	fig. 3		
oyster	*Bacillus* sp. *15.5814*	Gram-positive bacterium	Cg-BigDef1	*in vitro* activity tested against ecological microbes	De San Nicolas *et al*. [43]	their table 1		
cat	*Microsporum canis*	fungus	calprotectin	GWAS, polymorphism	Myers *et al*. [44]	paper topic		
human	atopic dermatitis	unknown cause, autoimmune	DEFB1	GWAS, polymorphisms common across two studies	Prado-Montes de Oca *et al*. [61]	paper topic	Ghareeb Mohamed *et al*. [62]	paper topic

In the realm of the microbiome, there are many individuals with difficult-to-treat dysbiosis-associated autoimmune syndromes such as diabetes and rheumatoid arthritis. While autoimmune damage is linked to disease symptoms, the underlying mechanisms initiating dysbiosis and autoimmune responses remain unclear [67]. Functional investigations of immune effectors as risk factors for dysbiosis, as done in *Drosophila* and *Hydra* [40,42], could reveal effector–microbe interactions that predispose individuals to dysbiosis not found through broad screens; indeed two loss-of-function mutations in fruit fly *DptA* actually *reduced* GWAS significance of the DptA S69R polymorphism by two orders of magnitude [37], emphasizing the need for careful consideration of exactly what kinds of signals can be recovered by different methodologies. Indeed, humans and other vertebrates encode similar genetic diversity in AMP genes [19], and focused studies of human beta-Defensin-1 have hit on two alleles that predict autoimmune atopic dermatitis [61,62], a disease associated with dysbiosis in the skin microbiome [68].

Finally, significant contributions of single effectors are a key consideration for agriculture. Many crop and animal species are relatively inbred, and could have inadvertently fixed for genetic variants of immune effectors that leaves them susceptible to pathogens. Of particular note are beneficial insects, both as pollinators (e.g. honeybees) and future food supplies (e.g. mealworms, black soldier flies) [69,70]. Ensuring these species have effective variants of key immune effectors for the microbes present in rearing conditions could make them more resilient to common infections.

## 7. Conclusion

Recent work on immune effectors of diverse animals suggests that the host immune system has evolved alongside its microbiome associates. This has equipped the host immune repertoire with specific effectors capable of controlling specific microbes. This finding revises decades-old assumptions about the nature of immune system evolution. However, to date this has been demonstrated most robustly in *Drosophila* and other invertebrates, which lack the antigen-antibody mechanisms of vertebrate adaptive immunity. We may owe the discovery of effector–microbe specificity to this unique trait of invertebrate models, as invertebrates lack adaptive immune responses, and so avoid this complicating signal in the data. That does not exclude that similar dynamics could underlie disease susceptibilities of vertebrates. Instead, it suggests a reason for why such specificities have not yet been discovered: because of the assumptions of the additive model of innate immune defence, the adaptive immune response was thought to be the level where specificity drove host–pathogen dynamics. The Achilles principle of immune evolution instead suggests that effectors can be key determinants of microbiome control, prophylactically preventing the need for the adaptive immune response to get involved. Importantly, the Achilles principle is driven by microbes having evolutionarily exploitable weaknesses. As the Achilles principle of immune evolution is driven by the microbes, not the host, I see no reason this concept should be restricted to invertebrates. Indeed, genetic variants in fruit fly and cat AMPs could explain common triggers of dysbiosis associated with chronic infections, perhaps overlapping with chronic inflammatory syndromes. This effector–microbe specificity that seems common in invertebrates could just be the tip of the iceberg in helping to explain the risk factors underlying many chronic and infectious diseases.

## Data Availability

This article has no additional data.
